# Pharmacokinetic-pharmacodynamic modelling of atazanavir in hair among adolescents on antiretroviral treatment in Zimbabwe

**DOI:** 10.1186/s40360-021-00497-8

**Published:** 2021-05-24

**Authors:** Bernard Ngara, Simbarashe Zvada, Tariro Dianah Chawana, Charles Fungai Brian Nhachi, Simbarashe Rusakaniko

**Affiliations:** 1 Department of Community Medicine, University of Zimbabwe College of Health Sciences, Mazowe Street, Parirenyatwa Complex, P. O Box A178 Avondale, Harare, Zimbabwe; 2 Department of Clinical Pharmacology, Stellenbosch University, Private Bag X1, Matieland, Stellenbosch, 7602 South Africa; 3 Department of Clinical Pharmacology, University of Zimbabwe College of Health Sciences, Mazowe Street, Parirenyatwa Complex, P. O Box A178 Avondale, Harare, Zimbabwe

**Keywords:** Pharmacodynamics, Pharmacokinetics, Modelling, HIV, Adolescents, Hair

## Abstract

**Background:**

Drug potency is a pharmacological parameter defining dose or concentration of drug required to obtain 50% of the drug’s maximal effect. Pharmacokinetic-pharmacodynamic modelling and simulation allows estimation of potency and evaluate strategies improving treatment outcome. The objective of our study is to determine potency of atazanavir in hair, defined as atazanavir level in hair associated with 50% probability of failing to achieve viral load below 1000 copies/ml among adolescents, and explore the effect of participant specific variables on potency.

**Methods:**

A secondary analysis was performed on data from a previous study conducted in HIV-infected adolescents failing 2nd line ART from Harare central hospital, Zimbabwe, between 2015 and 2016. We simulated atazanavir concentrations in hair using NONMEM (version 7.3) ADVAN 13, based on a previously established pharmacokinetic model. Logistic regression methods were used for PKPD analysis. Simulations utilising PKPD model focused on estimation of potency and exploring the effect of covariates.

**Results:**

The potency of atazanavir in hair was found to be 4.5 ng/mg hair before adjusting for covariate effects. Participants at three months follow-up, reporting adequate adherence, having normal BMI-for-age, and cared for by mature guardians had increased potency of atazanavir in hair of 2.6 ng/mg, however the follow-up event was the only statistically significant factor at 5% level.

**Conclusion:**

Atazanavir in hair in the range 2.6 to 4.5 ng/mg is associated with above 50% probability of early viral load suppression. Adherence monitoring to adolescents with lower potency of atazanavir is recommended. The effect self-reported adherence level, BMI-for-age, and caregiver status require further evaluation.

## Background

The application of exposure-response analysis remains very crucial in testing drug research or development strategies in all phases. The exposure may be the dose or a relevant pharmacokinetic (PK) measure such as drug concentration. The response is referred to as the pharmacodynamic (PD) outcome. If the exposure being used is derived from a PK model, such exposure-response analysis is also referred as PKPD modelling. Drug potency is the dose or concentration of a drug required to get 50% of that drug’s maximal effect, expressing the intensity of drug activity. A drug that causes a response to occur at a lower concentration has higher potency and is desirable compared to a drug causing the same response at a higher concentration [1].

Several models for exposure-response analysis estimate potency using simple to more complex modelling. Logistic regression is among the common methods used for PKPD modelling of binary response. This approach has been tested and advocated for use with the primary focus of determining the effective concentration corresponding to *x*-percent response (*EC*_*x*_) [2–7]. Although some findings show that the accuracy of *EC*_*x*_ estimates is questionable, the use of the logit model remains one of the robust methods to estimate the *EC*_*x*_ values and allows comparison of these values for different strategies being tested [8–10].

The effective concentration relating to concentrations that cause 50% of the maximum effect (*EC*_50_) defines the concentration at which the curvature of the response line occurs. The *EC*_50_ is a derived quantity estimated robustly, less dependent on the model accuracy, and is also a commonly used quantitative pharmacodynamic parameter in testing the potency of drug treatment strategy [11–16]. Additional parameters, such as *EC*_80_ or *EC*_90_ relating to concentrations that cause 80 and 90%, respectively, of the maximum effect are however also useful, but they highly depend on model accuracy [17].

Several studies showed that antiretroviral drug concentrations in hair predict virologic treatment outcome accurately. Some of these studies estimated antiretroviral concentrations in hair and thresholds associated with failing to suppress viral load derived using approaches which cannot meet the minimal acceptable standards of PKPD modelling [18–20]. The PKPD modelling approaches allow performance of simulation-based estimations and to derive strategies that optimize treatment outcome. Therefore, in this article, the aim is to establish a reference range for atazanavir concentration in hair associated with higher probability of failing to achieve viral load below 1000 copies/ml (i.e. potency) in adolescents for clinical practice. In doing so, the objective is to fit a PKPD model of the relationship between viral load and PK model-based simulated atazanavir concentration in hair, determine atazanavir in hair which corresponds to 50, 80 and 90% probabilities of treatment success, and additionally to explore the participant specific variables associated with a decrease in the EC_*50*_ value.

## Method

### Data source

Secondary analysis of data from a randomized clinical trial comprising 50 adolescents on atazanavir/ritonavir-based 2nd line antiretroviral therapy (ART) for at least 6 months was performed. In the primary study, participants were randomised to either modified directly administered antiretroviral therapy arm (intervention) or standard of care arm (control) and followed-up for 3 months. Informed consent questionnaires were used. Viral load and hair samples were collected at baseline and after 3 months follow-up [18]. Atazanavir concentrations in hair were determined using the liquid chromatography/mass spectrometry/mass spectrometry, with an assay range of 0.05–20 ng/mg. The trial is registered with the Pan African Clinical Trial Registry (PACTR201502001028169) and NIH Clinical Trials.gov (NCT02689895). The trial obtained ethical approval from local institutions which include the Joint Research Ethics Committee (JREC/51/14) and Medical Research Council of Zimbabwe (MRCZ/A/1840). Steady-state atazanavir concentrations in hair were simulated based previously established population PK model [21]. The PD outcome was the actual measured viral load dichotomised as below or above < 1000 copies/ml at three months follow-up. In this study, we maintained covariate effects that were significant in the population PK model.

### PKPD modelling

Simple logistic regression was used to fit a relationship between viral load below 1000 copies/ml and the simulated atazanavir concentration in hair as the baseline PKPD model. The probability of treatment failure (viral load ≥1000 copies/ml) for a given a hair concentration (X_1_) was estimated using the *“predict”* function in R-statistical package which applies to eq. 1, where β_0_ is the estimated regression constant, and β_1_ is the estimated regression coefficient for the X_1_ variable.

1 $$ \mathrm{Probability}\ \left(\mathrm{Failure}|{\mathrm{X}}_1\right)=\frac{{\mathrm{e}}^{\left[{\upbeta}_0+{\upbeta}_1{\mathrm{X}}_1\right]}}{1+{\mathrm{e}}^{\left[{\upbeta}_0+{\upbeta}_1{\mathrm{X}}_1\right]}} $$

We performed multivariate logistic regression to adjust for covariate effects on the relationship between viral load below 1000 copies/ml and the simulated atazanavir concentration in hair as the covariate PKPD model. Self-reported adherence, age-adjusted body mass index (BMI-for-age), the study follow-up event, and participant caregiver status were considered for testing based on contribution to the variability of atazanavir in hair reported earlier [18, 21]. Covariates were included in the full model if they exhibited the potential to decrease the *EC*_50_ value.

The probability of treatment failure was estimated for given: hair concentration level (*X*_1_), self-reported adherence measured by visual analogue scale (VAS) (*X*_2_), BMI-for-age (*X*_3_) categorised in three groups as underweight (<5th percentile), normal (5th to <95th percentile) and underweight (≥ 95th percentile), follow-up event (*X*_4_), and participant caregiver status (*X*_5_) using the *“predict”* function in R-statistical package which apply eq. 2, where β_0_ is the estimated regression constant, β_1_, β_2_, β_3_, β_4_ and β_5_ are the estimated regression coefficients for X_1_, X_2_, X_3,_X_4_ and X_5_ variables, respectively:

2 $$ \mathrm{P}\left(\mathrm{Failure}|{\mathrm{X}}_1;{\mathrm{X}}_2;{\mathrm{X}}_3;{\mathrm{X}}_4;{\mathrm{X}}_5\right)=\frac{{\mathrm{e}}^{\left[{\upbeta}_0+{\upbeta}_1{\mathrm{X}}_1+{\upbeta}_2{\mathrm{X}}_2+{\upbeta}_3{\mathrm{X}}_3+{\upbeta}_4{\mathrm{X}}_4+{\upbeta}_5{\mathrm{X}}_5\right]}}{1+{\mathrm{e}}^{\left[{\upbeta}_0+{\upbeta}_1{\mathrm{X}}_1+{\upbeta}_2{\mathrm{X}}_2+{\upbeta}_3{\mathrm{X}}_3+{\upbeta}_4{\mathrm{X}}_4+{\upbeta}_5{\mathrm{X}}_5\right]}} $$

Statistical significance of the covariates included in the final model and comparison of accuracy among hierarchical models were tested using analysis of variance (ANOVA) at 5% level of significance as part of model validation. Multicollinearity of the covariates included was checked using the mean variance inflation factor (VIF) value of less than 10 and there was no any relationship observed.

### Simulation and determination of ***EC***_***x***_ values

We simulated the relationship between viral load and atazanavir concentration in hair while adjusting for individual covariate effects. The participant specific variables that had a potential of decreasing the *EC*_50_ value were used in simulation of the full covariate PKPD model. Simulations were performed using the using the *“predict”* function in R-statistical package. Plot diagrams of simulated concentration versus probability of treatment failure including 95% confidence interval (CI) represented by shaded areas were constructed using the *“ggplot2”* functions in R-statistical package. Graphical interpolation was performed on the plots to determine baseline *EC*_*x*_ values and also to present the effect of individual and multiple covariates on the *EC*_50_ value.

## Results

### Description of study data

The mean age was 15.8 years (standard deviation – 1.8) and 54% were females. In terms of education, 89% had completed secondary school, 9% completed primary school and 2 were dropouts. About 54% had normal BMI, 15% were overweight while 30% were underweight. Regards to caregivers, 20% were biological parents, 40% were grandparents, 10% were siblings and 30% were aunt or uncle. About 32% were in the early WHO disease progression stage at ART initiation, while 68% were of the late stage. The mean adherence by visual analogue scale (VAS) score was 84.2% (standard deviation – 18.1). The median length of hair was 1 cm (range – 0.5 cm to 1.5 cm). The mean hair weight was 2 mg (standard deviation – 0.15). Most of the study participants estimated to be 82% were on tenofovir/lamivudine/atazanavir/ritonavir drug combination. The mean (95% confidence interval) simulated atazanavir concentration in hair was 1.2 (0.8–1.6) ng/mg hair and 2.2 (1.5–2.8) ng/mg hair at enrolment and follow-up, respectively. The median (interquartile range) of observed viral load was 70,676 (21,862 to 164,800) copies/ml and 8141 (< 40 to 82,250) copies/ml at enrolment and follow-up, respectively. All participants were adolescents with virologic treatment failure (viral load ≥1000copies/ml) to atazanavir-based 2nd line ART at enrolment. About 40% of the study participants became virologically suppressed (viral load <1000copies/ml) after follow-up.

### Baseline ***EC***_**50**_, ***EC***_**80**_ and ***EC***_**90**_ values

**Fig. 1:**
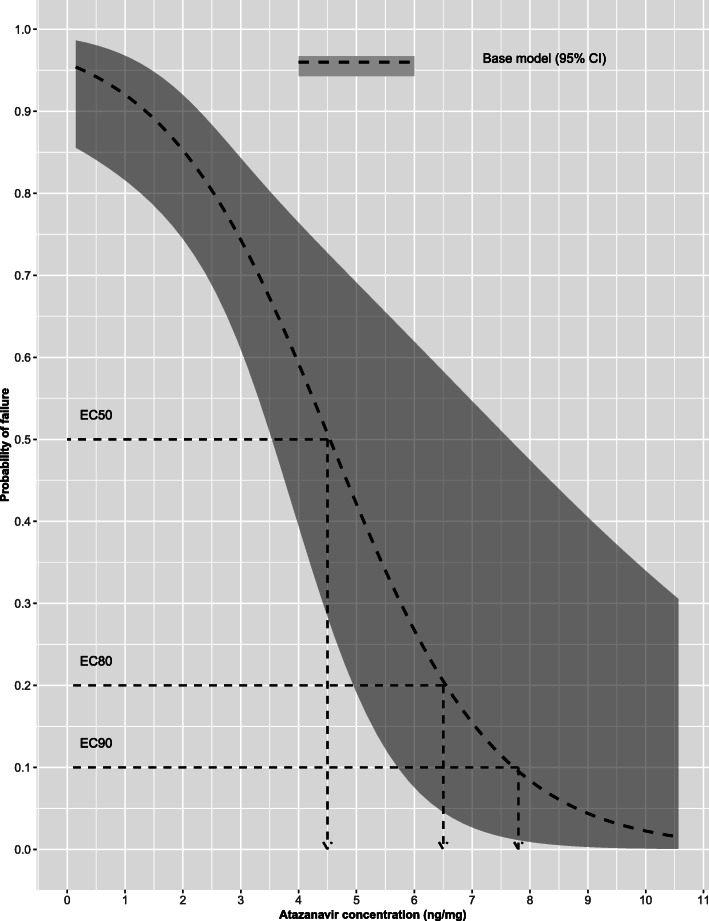
Exposure–response curve presenting the baseline relationship between probability of failing to achieve viral load suppression (dashed line) including 95% confidence interval (shaded ribbon) and the simulated atazanavir concentrations in hair. The baseline *EC*_50_, *EC*_80_ and *EC*_90_ values are illustrated on the plot (dashed arrows)

The *EC*_50_ of atazanavir in hair was estimated to be 4.5 ng/mg hair with a 95% confidence interval of 3.5 to 7.6 ng/mg hair. The *EC*_80_ was estimated to be 6.5 ng/mg and a right-tailed 95% confidence interval with a lower limit of 4.9 ng/mg hair, while the *EC*_90_ was estimated to be 7.8 ng/mg hair and a right-tailed 95% confidence interval with a lower limit of 7.8 ng/mg hair. Figure 1 illustrates further the relationship between probability of failing to achieve viral load below 1000 copies/ml and the simulated atazanavir concentrations in hair.

### Effect of self-reported adherence on ***EC***_**50**_ value

**Fig. 2:**
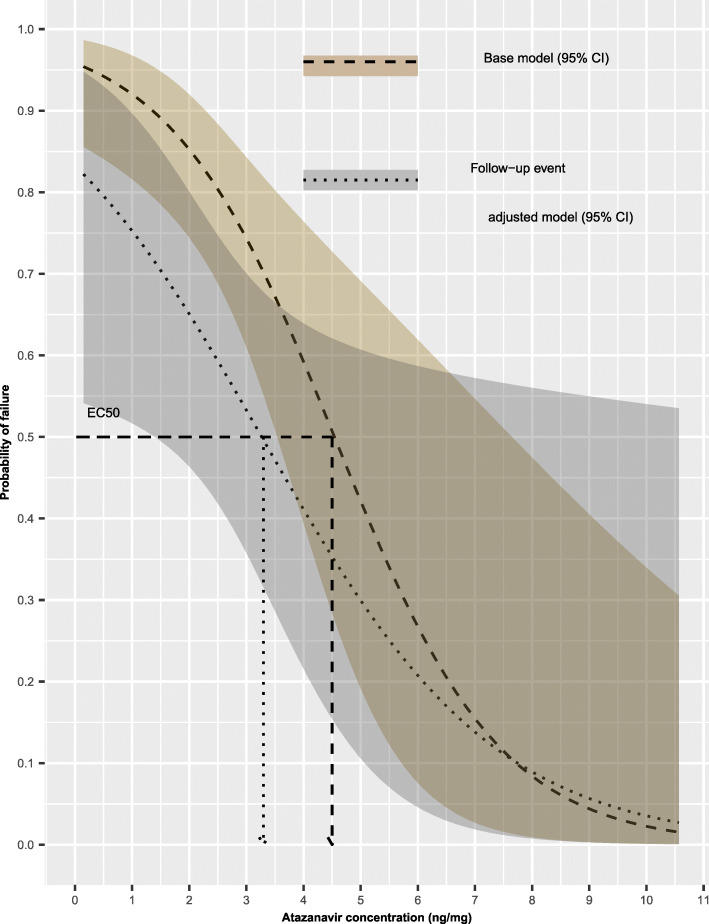
Exposure–response curves presenting the baseline relationship between probability of failing to achieve viral load suppression (dashed line) including 95% confidence interval (shaded ribbon) and the simulated atazanavir concentrations in hair, comparing with the effect of 50% self-reported adherence to the relationship between probability of failing to achieve viral load suppression (dotted line) including 95% confidence interval (shaded ribbon) and the simulated atazanavir concentrations in hair. The baseline and 50% self-reported adherence adjusted *EC*_50_ values are illustrated on the plot (dashed and dotted arrows, respectively)

The average *EC*_50_ for a participant with 100% self-reported adherence was estimated to be approximately equal to 4.5 ng/mg hair and a 95% confidence interval of 3.5 to 7.6 ng/mg hair. The average *EC*_50_ for a participant with 50% self-reported adherence was estimated to be approximately equal to 4.4 ng/mg hair and a right-tailed 95% confidence interval with a lower limit of 1.8 ng/mg hair. The effect of 50% self-reported adherence on the relationship between viral load and the simulated atazanavir concentrations in hair is illustrated further in Fig. 2.

### Effect of BMI-for-age on ***EC***_**50**_ value

**Fig. 3:**
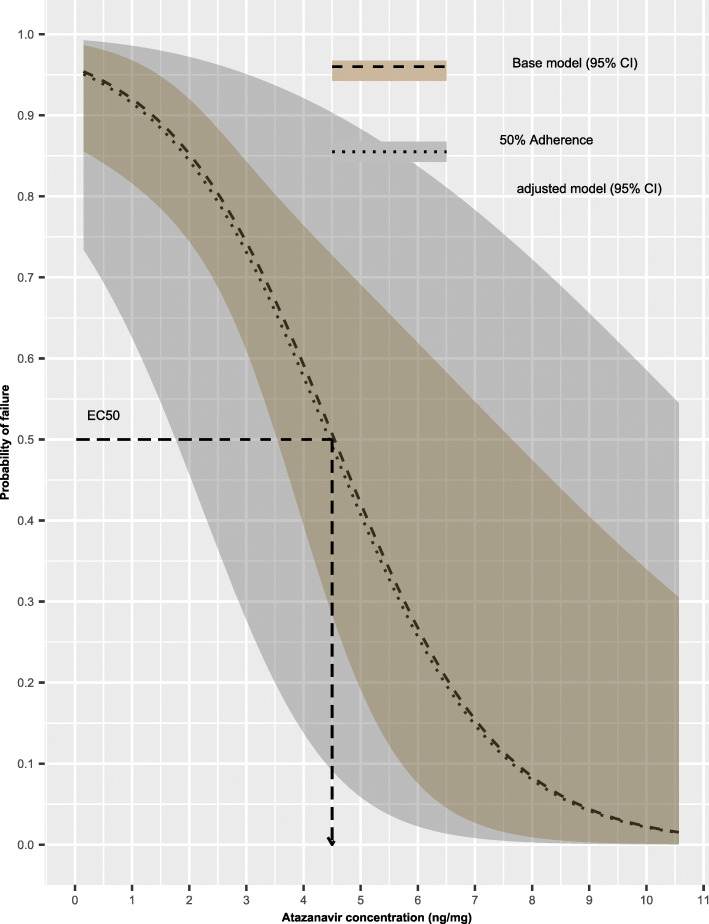
Exposure–response curves presenting the baseline relationship between probability of failing to achieve viral load suppression (dashed line) including 95% confidence interval (shaded ribbon) and the simulated atazanavir concentrations in hair, comparing with the effect of normal BMI-for-age to the relationship between probability of failing to achieve viral load suppression (dotted line) including 95% confidence interval (shaded ribbon) and the simulated atazanavir concentrations in hair. The baseline and normal BMI-for-age adjusted *EC*_50_ values are illustrated on the plot (dashed and dotted arrows, respectively)

The average *EC*_50_ for normal BMI-for-age participants was estimated to be approximately 3.8 ng/mg hair and a 95% confidence interval of 2.7 to 6.8 ng/mg hair compared to that for participants with normal BMI-for-age. The average *EC*_50_ for overweight participants was estimated to be approximately 3.5 ng/mg hair and a 95% confidence interval of 1.8 to 7.5 ng/mg hair compared to that for participants with normal BMI-for-age. The average *EC*_50_ for overweight participants was estimated to be approximately 5.5 ng/mg hair and a right-tailed 95% confidence interval with a lower limit of 3.5 ng/mg hair compared to that for participants with normal BMI-for-age. Figure 3 shows further the effect of normal BMI-for-age to the relationship between viral load and the simulated atazanavir concentrations in hair.

### Effect of caregiver status on ***EC***_**50**_ value

**Fig. 4:**
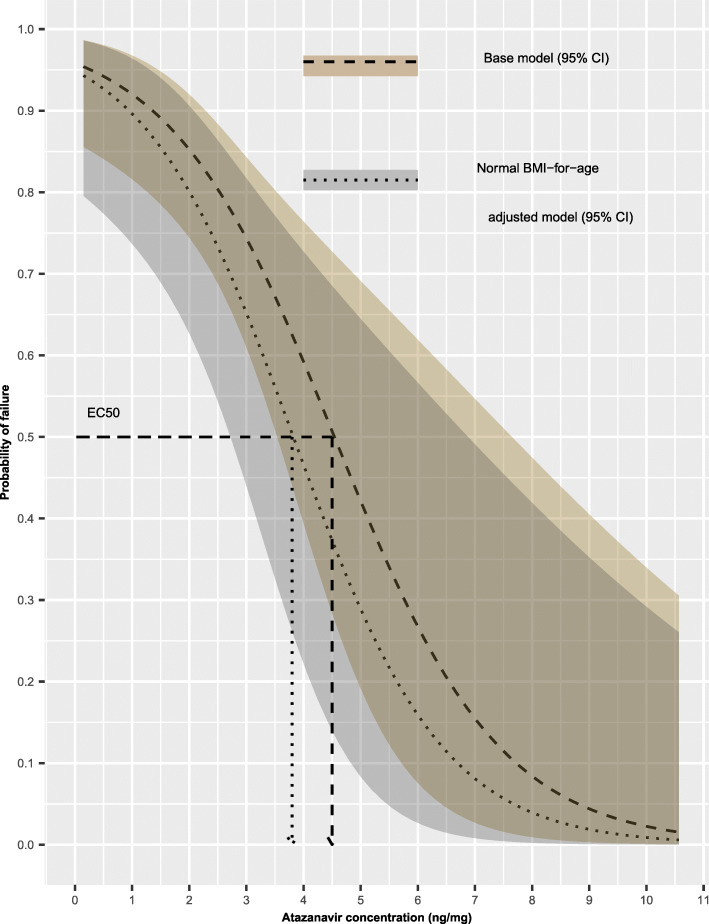
Exposure–response curves presenting the baseline relationship between probability of failing to achieve viral load suppression (dashed line) including 95% confidence interval (shaded ribbon) and the simulated atazanavir concentrations in hair, comparing with the effect of mature care-giver status to the relationship between probability of failing to achieve viral load suppression (dotted line) including 95% confidence interval (shaded ribbon) and the simulated atazanavir concentrations in hair. The baseline and the mature care-giver status adjusted *EC*_50_ values are illustrated on the plot (dashed and dotted arrows, respectively)

We estimated that the *EC*_50_ for participants who received care from mature caregivers (≥ 19 years of age) approximately decreased to 3.8 ng/mg hair and 95% confidence interval of 2.3 to 6.4 ng/mg hair compared to that for participants receiving care from younger siblings (10 to 18 years). Figure 4 shows further the effect of receiving care from mature caregivers to the relationship between viral load and the simulated atazanavir concentrations in hair.

### Effect of the follow-up event status on ***EC***_**50**_ value

**Fig. 5:**
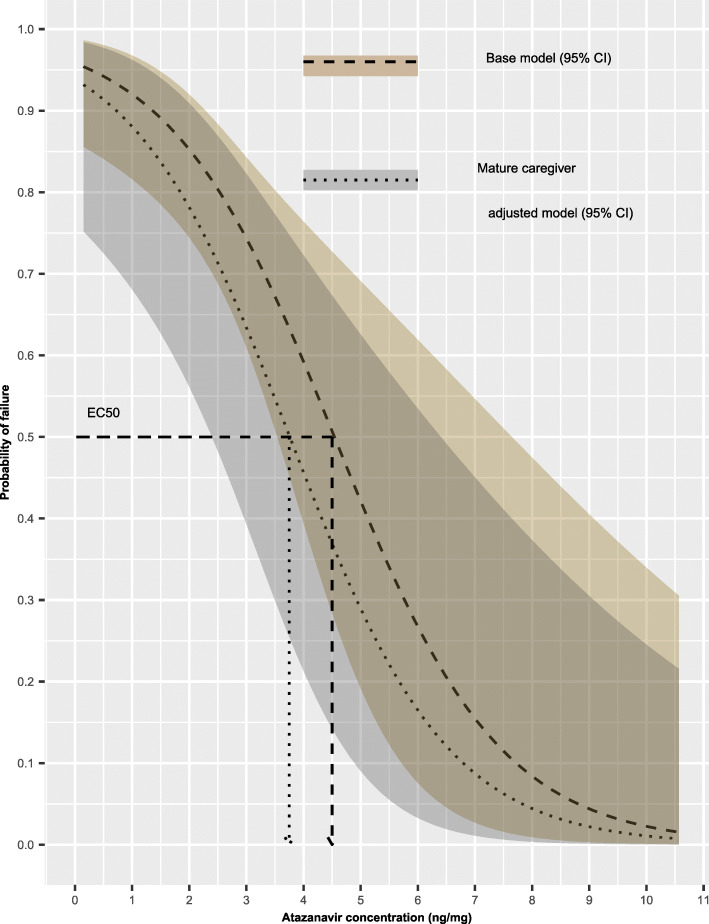
Exposure–response curves presenting the baseline relationship between probability of failing to achieve viral load suppression (dashed line) including 95% confidence interval (shaded ribbon) and the simulated atazanavir concentrations in hair, comparing with the effect of the follow-up event to the relationship between probability of failing to achieve viral load suppression (dotted line) including 95% confidence interval (shaded ribbon) and the simulated atazanavir concentrations in hair. The baseline and the follow-up event adjusted *EC*_50_ values are illustrated on the plot (dashed and dotted arrows, respectively)

The *EC*_50_ at the 3-months follow-up occasion was estimated to be an average of 3.3 ng/mg hair and right-tailed 95% confidence interval with a lower limit of 1.4 ng/mg hair compared to that for the same participants at enrolment, irrespective of the study arm for these subjects. Figure 5 illustrates further the effect of the follow-up event to the relationship between viral load and the simulated atazanavir concentrations in hair.

### Effect of multiple covariates on ***EC***_**50**_ value

**Fig. 6:**
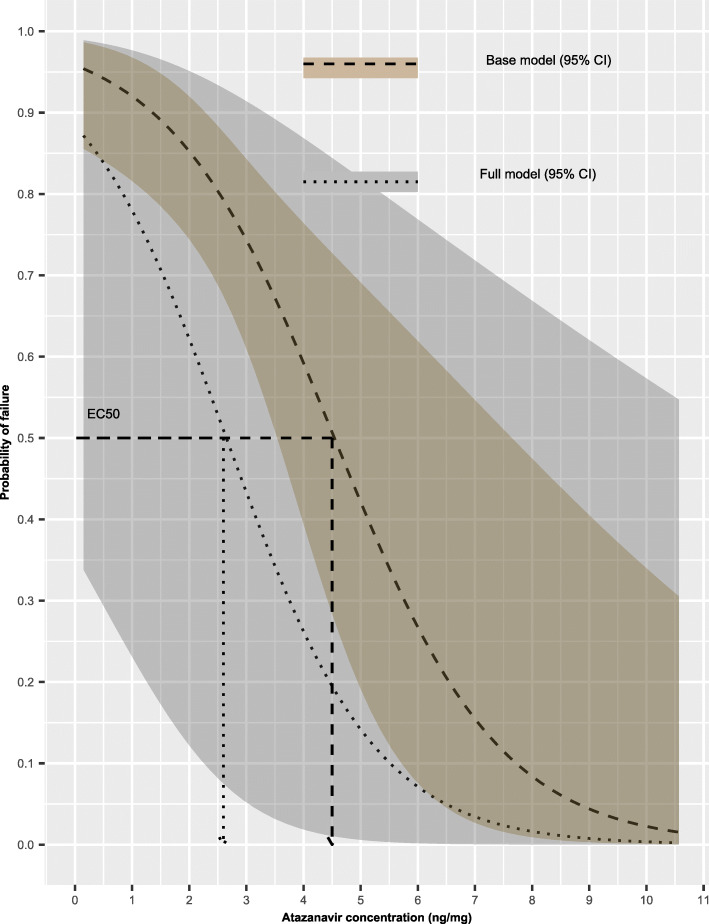
Exposure–response curves presenting the baseline relationship between probability of failing to achieve viral load suppression (dashed line) including 95% confidence interval (shaded ribbon) and the simulated atazanavir concentrations in hair, comparing with the effect of the 3-months follow-up occasion, reporting adequate adherence, having normal BMI-for-age, and receiving care from mature caregivers concurrently (dotted line) including 95% confidence interval (shaded ribbon) and the simulated atazanavir concentrations in hair. The baseline and the follow-up event adjusted *EC*_50_ values are illustrated on the plot (dashed and dotted arrows, respectively)

The *EC*_50_ for these adolescents at 3-months follow-up occasion, reporting adequate adherence, normal BMI-for-age, and receiving care from mature caregivers concurrently was estimated to be approximately 2.6 ng/mg hair and left-tailed 95% confidence interval with an upper limit of 5.5 ng/mg hair. Figure 6 illustrates further the effect of the multiple covariates on the relationship between viral load and the predicted atazanavir concentrations in hair.

### Statistical significance of covariates on atazanavir potency

The 3-months follow-up occasion reduced the *EC*_50_ value significantly at 5% level (*p* < 0.001), while all the other covariates which were included in the full model were not statistically significant at 5% level. Additional information for the effect of individual covariate and the multiple covariates concurrently (full model) are presented in Table 1.

**Table 1:** Covariate effects on *EC*_50_ values

Adjusted ***EC***_**50**_ value	Estimated ***EC***_**50**_ in ng/mg of atazanavir in hair (95% Confidence Interval)	***p***-value
***EC***_**50**_: Base model	4.5 (3.5–7.6)	(NA -reference model)
***EC***_**50**_: Follow-up event	3.3 (1.4 - □)	< 0.001
***EC***_**50**_: 100% Self-reported adherence	4.5 (3.5–7.6)	0.304
***EC***_**50**_: 50% Self-reported adherence	4.5 (1.8 – □)	0.304
***EC***_**50**_: Normal BMI-for-age participants	3.8 (2.7–6.8)	0.927
***EC***_**50**_: Overweight participants	3.5 (1.8–7.5)	0.927
***EC***_**50**_: Underweight participants	5.5 (3.5 – □)	0.927
***EC***_**50**_: Mature care-givers	3.8 (2.3–6.4)	0.636
***EC***_**50**_: Full model	2.4 (□ – 5.5)	0.919

## Discussion

We performed the population analysis to explore the relationship between atazanavir concentrations in hair and virologic response in adolescents failing 2nd line ART through the development of logistic regression models. The exposure of interest used in this work was simulated atazanavir concentrations based on our earlier work [21]. Some previous work showed that mathematical modelling can improve estimation of *EC*_50_ when steady-state simulations from a mathematical model are used, hence it will be possible to interpret previously obtained datasets again, and also to obtain accurate estimates of *EC*_50_ even under circumstances where steady-state measurements are not experimentally feasible [22].

The choice of using atazanavir in the model does not have an association with the usefulness of drugs in clinical practise, but merely because they were feasibly available to the authors during the modelling work. Although the modelling results in this article add some knowledge for clinical practise, a particular interest was also to demonstrate a modelling framework usable to predict treatment outcome based on a measure of drug exposure obtained from hair. This approach can be extrapolated or adopted for use to any other drug being used in clinical practise. Drug exposure in hair has been found to predict treatment outcomes better than any standard measures known [23]. The fact that the PKPD model has a dose component in its structural mechanism, a key component to determine the need for dose individualisation influenced the choice of the model framework. However, evaluation of dose-adjustment could not be performed in the current analysis because of the low sample size (such that *n* < 30 for different categories of the BMI-for-age).

The study 3-months follow-up occasion was the only covariate that significantly improved potency of atazanavir in hair at 5% level of significance irrespective of the study arm. This could be that by taking part in the clinical trial, the participants had the knowledge that they were under study, possibly influencing positively their adherence to treatment and hence increasing their chances of treatment success. There was no blinding involved in the primary study during randomization of study participants to study arms [24], hence increasing the possibilities of bias. The covariates self-reported adherence, BMI-for-age, and participant caregiver status were included in the final model even though they were not significant at 5% level of significance, but because they reduced the *EC*_50_ of atazanavir concentrations in hair clinically significantly. Hence the individual effect of these covariates needs further evaluation in the future studies.

The simulations performed show that probabilities of antiretroviral treatment success among those reporting 50% or 100% self-reported adherence is the same. However participants with 50% self-reported adherence had wider confidence intervals compared to participants with 100% self-reported adherence which accurately predicted treatment success the same as atazanavir concentration in hair. This finding supports earlier findings which showed that protease inhibitor levels in hair predict virologic treatment outcome better than self-reported adherence which provide information on exposure to a drug of interest over a short period and is affected by recall bias [19, 20, 25].

Participants with normal BMI-for-age improved potency of atazanavir in hair while being either overweight or underweight reduced potency during multivariate analysis. This also concurs with earlier findings which reported that normal to higher BMI is associated with improved immunological health compared to lower BMI [26–28]. Participants receiving care from mature caregivers have increased chances of viral load suppression, again supporting findings from previous studies. Their findings highlighted the effect of caregiver maturity on psychological distress to the complexity of perinatal HIV-infection, chronic HIV disease and lifelong ART in adolescents [29, 30].

The *EC*_50_ of atazanavir in hair for adolescents was estimated to be 4.5 ng/mg hair before adjusting for covariates effect. However the *EC*_50_ of atazanavir in hair was optimally reduced to 2.4 ng/mg hair when the model was simulated using participant specific variables that had potential to reduce the value. The model-based optimal estimate of the *EC*_50_ of atazanavir in hair concurs with the result reported in the primary study which proposed a cut-off of 2.35 ng/mg, the lower quartile of the observed atazanavir concentration in hair among participants who achieved viral load suppression at 3-months follow-up [18].

To the best of our knowledge, this is the first study to apply mathematical modelling to establish cut-off points for antiretroviral drugs measured in hair which are associated with certain levels of the probability of viral load suppression. Some covariates were reported in both the bivariate and multivariate models on the basis of their clinical significance even though they were not statistically significant. In this study, our 95% confidence intervals of parameter estimates may look higher, which can be attributed to the fact that both the PK [21] and the PKPD modelling was based on a limited and small sample size (*n* = 50). Additionally, most of the simulated atazanavir in hair concentrations were below the *EC*_50_, *EC*_80_ and *EC*_90_ values based on the model projections which is a sign of model over-prediction, something which could have been influenced again by the limited sample sizes It is important both future primary and modelling studies which are going to be conducted to collect a wide range factors that influence exposure-response and also use a large sample size.

## Conclusion

In conclusion, atazanavir in the hair in the range 2.4 to 4.5 ng/mg is likely associated with at least50% chances of HIV suppression. Hair drug concentration predicts chances of viral suppression more accurately when compared to self-reported adherence. Direct monitoring of dose ingestion is recommend for adolescents failing 2nd line ART to improve chances of viral suppression. Also specialised health-care interventions are recommended for adolescents on ART whose caregivers are immature to improve their chances of viral suppression. Dose-adjustment require to be evaluated further especially for underweight or overweight participants using PKPD modelling.

## Data Availability

The primary study did not publish the data to the public, however the data can be available upon request and approval from the principal investigators of the primary study. The principle investigator of the primary study was Tariro Dianah Chawana, and can be contacted on email (tdchawana@gmail.com).
